# Evaluating the Clinical Impact of a Polyphenol-Rich Extract from *Salicornia ramosissima* on Patients with Transient Ischemic Attack and Minor Stroke

**DOI:** 10.3390/nu16244307

**Published:** 2024-12-13

**Authors:** Ana M. Nájar, Cristina López Azcárate, Carmen Domínguez Ruiz, David Núñez-Jurado, Reyes de Torres, Reyes López, Miriam Camino-Moya, Eleonora Magni, Emilio Montero-Ramirez, Antonio Bocero, Álvaro Laviana, Teresa Busquier Cerdán, Antonio León, Carmen del Rio, Joan Montaner, Soledad Pérez-Sánchez

**Affiliations:** 1 Instituto de Biomedicina de Sevilla, IBiS/Hospital Universitario Virgen Macarena/CSIC/Universidad de Sevilla, 41013 Seville, Spain; anmoyano@us.es (A.M.N.); lopezcristinafisevi@gmail.com (C.L.A.); carmendominguezneuro@gmail.com (C.D.R.); david.nunez.jurado.sspa@juntadeandalucia.es (D.N.-J.); reyesdetorreschacon@gmail.com (R.d.T.); miriam.cam.moy@gmail.com (M.C.-M.); magni@us.es (E.M.); jmontaner-ibis@us.es (J.M.); soledad.perez.sanchez@gmail.com (S.P.-S.); 2 Department of Biochemistry, Hospital Universitario Virgen Macarena, 41009 Seville, Spain; antonio.leonj.sspa@juntadeandalucia.es; 3 Department of Psychology and Human Neuroscience, Universidad Loyola Andalucía, 41704 Seville, Spain; rlopezjimenez@al.uloyola.es; 4 Research Group CTS969: “Care Innovation and Health Determinants”, Faculty of Nursing, Physiotherapy and Podiatry, University of Seville, 41004 Sevilla, Spain; 5 Department of Neurology, Hospital Universitario Virgen Macarena, 41009 Seville, Spainanboga96@gmail.com (A.B.); alvarolavianamarin@gmail.com (Á.L.); 6 Department of Radiology, Hospital Universitario Virgen Macarena, 41009 Seville, Spain; tbuscer@gmail.com; 7 Instituto de Biomedicina de Sevilla, IBiS/Hospital Universitario Virgen del Rocío/CSIC/Universidad de Sevilla, 41013 Seville, Spain; 8 Neurovascular Research Laboratory, Vall d’Hebron Institute of Research (VHIR), Hospital Vall d’Hebron, 08035 Barcelona, Spain

**Keywords:** Salicornia, transient ischemic attack, minor stroke, polyphenols, vascular risk, homocysteine

## Abstract

Transient ischemic attack (TIA) is a well-established risk factor for future strokes, making interventions that target recovery and vascular risk crucial. This study aimed to assess the safety and clinical effects of a polyphenol-rich *Salicornia ramosissima* extract in post-TIA patients. A randomized, triple-blind, placebo-controlled trial was conducted with participants who had a history of TIA or minor stroke and who received 1 g of Salicornia extract or placebo over 11 months. Biochemical analyses, neuropsychological assessments (MOCA test), and gait and aerobic performance tests were conducted at the beginning and the end of the study. A total of 118 individuals were screened, with 80 finally included. Importantly, no significant adverse events were reported throughout the study. A neurological analysis showed an improvement in MOCA scores in patients treated with the Salicornia extract for 11 months. The treatment did not affect spatiotemporal gait parameters, but it significantly reduced blood pressure at baseline and after the aerobic performance test. Biochemically, both groups exhibited mild hyperhomocysteinemia at baseline; however, Salicornia treatment significantly lowered homocysteine levels, bringing them within the normal range. These findings highlight the safety of the Salicornia extract in patients at a high cerebrovascular risk and suggest it as a potential therapeutic option for managing vascular risk factors, such as hyperhomocysteinemia and hypertension. However, further studies are required to confirm the underlying mechanisms and explore broader clinical applications.

## 1. Introduction

Stroke is the second leading cause of death worldwide and the third leading combined cause of death and disability in adulthood [1]. There are two main subtypes of stroke: hemorrhagic and ischemic stroke, caused by the rupture or the occlusion of a blood vessel by a thrombus, respectively. Both types result in the localized deprivation of oxygen and nutrients, leading to the death of brain cells in the affected area [2]. Approximately 85% of strokes are ischemic [3].

A transient ischemic attack (TIA) is clinically defined as the acute onset of focal neurological symptoms that resolve completely without causing acute infarction or tissue damage on neuroimaging. If a lesion is present, it is classified as a minor stroke [4]. The symptoms of TIA are mild but may be considered a serious warning of impending ischemic stroke. Therefore, they should be evaluated immediately to determine the underlying cause and initiate appropriate therapeutic interventions to reduce the risk of a subsequent ischemic stroke [5,6]. These interventions should focus on managing modifiable risk factors, such as hypertension, diabetes, dyslipidemia, smoking, unhealthy dietary habits, low physical activity, and a high body mass index (BMI) [1].

Research suggests that a healthy lifestyle, with diet as a crucial factor, can reduce the risk of stroke by approximately 80% [7]. It has also been reported that adherence to a high-quality diet can lower the risk of a first stroke by 40%, regardless of other risk factors. Conversely, a poor-quality diet has been strongly associated with an increased risk of stroke [8]. The protective health effects associated with healthy dietary patterns, which include a high consumption of fruits and vegetables, are largely due to the polyphenols contained in these plant-based foods [9]. Polyphenols have demonstrated robust antioxidant and anti-inflammatory capacities, as well as antihypertensive or anti-atherosclerotic effects, among others. With more than 40 types of biological activities, polyphenols can modulate different processes involved in the development of a wide range of diseases, including stroke. As a result, polyphenols hold a unique place in the search for natural compounds with pharmacological properties that support and maintain health [10,11].

The synthesis and accumulation of polyphenols in plants is part of their adaptive response to stress situations. *S. ramosissima* is an annual halophyte plant that grows spontaneously in coastal areas and marshes in Europe, particularly in Spain and Portugal [12]. Research highlights that *S. ramosissima* has significant nutritional and functional properties with potential health benefits [13]. More than 60 polyphenolic compounds have been identified in *S. ramosissima*, with a profile predominantly consisting of flavonoids and phenolic acids. Notable compounds include well-known molecules, such as quercetin, epicatechin, and luteolin, as well as less-studied, plant-specific compounds, like caffeoylquinic acids and tungtungmadic acid. Importantly, the polyphenol content of *S. ramosissima* is not static; it varies significantly in response to environmental factors, such as salinity and other stress conditions. In vivo studies have drawn attention to the neuroprotective effects of the polyphenolic compounds found in *S. ramosissima* in models of cerebral ischemia [14]. Building on this evidence, the combination of these polyphenols, acting across multiple levels and molecular pathways, represents promising potential for stroke prevention and treatment. In our laboratory, we explored the effects of polyphenol-rich extracts of *S. ramosissima* in animal models of cerebral ischemia, demonstrating its neuroprotective potential [15]. Other preclinical studies using extracts from various species of Salicornia have also investigated their neuroprotective effects [16,17], as well as their benefits on cardiovascular risk factors, such as obesity [18], dyslipidemia [19], hyperglycemia [20], or vascular remodeling [21]. Recently, we reported that dietary supplementation with *S. ramosissima* extracts in healthy subjects for three months was safe and increased the glomerular filtration rate, reduced HDL-cholesterol, and modulated plasma markers related to cardiovascular disease [22].

While these preclinical findings are promising, large randomized clinical trials of dietary interventions in individuals who have already suffered a stroke are needed to provide evidence for preventing recurrence [23]. In this regard, it is important to note that risk factors, such as hypertension and dyslipidemia, can be modulated through diet, both in primary and secondary prevention strategies [24]. In this sense, several clinical trials have reported the safety and benefits of the supplementation with different polyphenol-rich extracts in diseases, such as stroke [25,26], and its associated risk factors [27,28,29,30,31,32]. However, we are aware of only one clinical study that reported the safety of consuming 600 mg/day of *Salicornia europaea* L. extracts in subjects complaining of memory dysfunction. Although the study did not show significant improvements in the primary outcome, which was cognitive performance, these findings underscore the need for further clinical trials to gather more robust evidence [33]. *S. ramosissima* is a promising source of polyphenols with neuroprotective, anti-inflammatory, and antihypertensive properties [14]. However, its potential in clinical contexts, particularly for secondary stroke prevention, remains underexplored. The reported benefits of *S. ramosissima* extracts on cardiovascular risk factors, coupled with its safety profile in healthy subjects, make it an ideal candidate for investigations in high-risk populations, such as those who have experienced a TIA or minor stroke.

This study aims to address this gap by evaluating the safety of Salicornia extract supplementation in individuals who had experienced a TIA or minor stroke. We also assessed the efficacy of the extracts in preventing new cardiovascular events and explored their potential effects on blood parameters, cognitive function, and gait impairment. By focusing on a high-risk population, this work represents a critical step toward translating preclinical evidence into practical dietary strategies for secondary stroke prevention.

## 2. Materials and Methods

### 2.1. Study Design and Ethics Statement

This randomized, triple-blind, placebo-controlled, parallel-group clinical trial aimed to evaluate the safety and neurovascular-protective potential of a polyphenol-rich *S. ramosissima* extract in patients who had suffered a transient ischemic attack (TIA) or minor stroke. Patients were randomized in a 1:1 ratio to receive either *S. ramosissima* extract or a placebo over a period of 11 months, with clinical assessments at three key points: baseline (visit 1), 6 months (visit 2), and at the study’s conclusion (visit 3) at 11 months.

This study was conducted at the Virgen Macarena University Hospital (Seville, Spain) from October 2022 to June 2024, in accordance with the principles of the Declaration of Helsinki [34] and in the Standards of Good Clinical Practice ICH E6 [35]. The trial was pre-registered in the US National Library of Medicine’s Clinical Trials Registry and Results Database (clinicaltrials.gov) under the identifier NCT06076122. Ethical approval was obtained from the Andalusian Ethics Committee of Biomedical Research (ID HALOFITAS; PEIBA code 0371-M2-23) on 18 February 2022, ensuring the highest standards of ethical conduct and participant safety. This manuscript adheres to the applicable Consolidated Standards of Reporting Trials (CONSORT) guidelines.

### 2.2. Investigational Product and Blinding

The study had two treatment arms in a 1:1 ratio: Salicornia from Hydroponia (SH) extracts and a placebo. *S. ramosissima* plants were grown hydroponically in Faro, Portugal [https://riafresh.com/, accessed on 1 September 2024]. Extracts were manufactured by EVESA (Cadiz, Spain [https://evesa.com/, accessed on 1 September 2024]) from the fresh aerial parts of the plant following a drying process. To ensure adequate masking, BIO-DIS Laboratories (Seville, Spain [https://www.bio-dis.com/, accessed on 1 September 2024]) prepared capsules of Salicornia extracts and placebo that were physically identical.

The capsules were stored in bottles labeled with a unique coding for each participant. Each coding corresponded randomly and indistinguishably to the SH extract or placebo. The list indicating which treatment corresponds to each coding was sent to the Pharmacy Service of the hospital and was only revealed to the research team after completing the main analysis of the results, maintaining triple blinding. 

### 2.3. Participant Recruitment 

Between October 2022 and July 2023, a total of 80 patients who previously attended the Virgen Macarena University Hospital were recruited via telephone. During these calls, participants received a brief overview of the study’s purpose, the selection criteria, and their potential role as participants.

Inclusion criteria were adults aged ≥ 18 years with typical stroke symptoms lasting less than 24 h classified as TIA or minor stroke (if diffusion-weighted imaging was positive on magnetic resonance imaging) during the last 24 months and who had neuroimaging performed at the time of the acute episode that ruled out other non-vascular lesions. Eligible patients also needed to be willing and able to provide informed consent.

Patients who met any of the following criteria were excluded through a pre-study medical evaluation: (i) use of nutritional supplements containing vitamins or polyphenols within 30 days prior to screening, (ii) participation in any other clinical trial involving medications within 30 days prior to screening, or plans to do so during the study, (iii) presence of hyperthyroidism, (iv) dysphagia, (v) dependence in basic activities of daily living (modified Rankin Scale score > 3), (vi) known allergies or intolerance to halophyte plants, (vii) the regular consumption of halophyte plants, (viii) pregnancy or breastfeeding, (ix) active neoplastic disease, or (x) any serious illness with a life expectancy of less than 12 months.

Once eligibility was verified and after signing the informed consent, the patients were randomly assigned with a unique coding. Participants were instructed to take two capsules daily for eleven months, containing a total of 1 g of SH extracts or placebo according to the study arm.

### 2.4. Safety Outcome Collection

To assess the safety and tolerability of the supplement, the incidence of adverse events (AEs) was systematically collected during in-person visits (visits 2 and 3) and through intermediate remote contacts facilitated by the study’s dedicated telephone number provided to participants. AEs were defined as any untoward medical occurrences occurring during the study period.

All reported AEs were documented in the participants’ medical records and recorded on the Case Report Form (CRF). Each AE was categorized according to the Medical Dictionary for Regulatory Activities (MedDRA, version 25.0) and organized into System Organ Classes (SOC). Furthermore, AEs were classified based on causality (relationship with treatment) and severity using medical judgment. Severity criteria included death, life-threatening events, hospitalization, prolonged hospital stays, permanent or major disability or incapacity, and other medically significant serious AEs (SAEs).

Only AEs potentially related to the treatment were analyzed in detail. The frequency of AEs and the percentage of AEs leading to participant withdrawal from the study were determined.

### 2.5. Lifestyle Questionnaires

At baseline (visit 1), participants’ dietary habits were assessed using two validated instruments: the 14-item Mediterranean Diet Adherence Questionnaire (MEDAS) and the 137-item Food Frequency Questionnaire (FFQ) developed for the PREDIMED study. Physical activity levels were evaluated using the short version of the 7-item International Physical Activity Questionnaire (IPAQ).

### 2.6. Cognitive and Gait Assessment

A cognitive and gait assessment was performed for all patients in the study before (visit 1) and after eleven months (visit 2) of dietary intervention. These evaluations were performed by a qualified neuropsychologist and physiotherapist, respectively, blinded to the patient’s allocation to ensure accurate and reliable assessments. 

The Spanish version of the Montreal Cognitive Assessment (MoCA) [36] was used as a screening tool to evaluate the cognitive functions of participants. This validated instrument is widely employed to detect cognitive impairment. The MoCA assesses various cognitive domains, including attention, memory, language, visuospatial abilities, and executive functions. 

The gait assessment protocol included the following procedures: measurement of gait speed and Functional Ambulation Performance (FAP) using GAITRite^®^ equipment (CIR Systems Inc., Franklin, NJ, USA), measured before and after the Six minutes Walking Test (6MWT) [37]; assessment of the aerobic capacity and endurance using 6MWT; measurement of systolic blood pressure (SBP), diastolic blood pressure (DBP), and heart rate (HR) using ProBP Digital Blood Pressure Device (Welch Allyn, New York, NY, USA), before, immediately after, and at 1 and 5 min after. Balance was evaluated using the Berg Balance Scale [38], which consists of 14 items designed to assess postural control, as well as both static and dynamic balance. Strength was measured in the upper limbs (hand strength) using a manual dynamometer (Smedley, Takei Scientific Instruments Co., Nigata, Japan).

### 2.7. Blood Analysis

Analytical studies were performed at baseline (visit 1), visit 2, and 3. After an overnight fast, venous blood was collected (between 8 a.m. and 11 a.m.): one in a tube with an ethylenediaminetetraacetic acid (EDTA) anticoagulant (BD Vacutainer K2EDTA spray-coated tubes) for plasma cell counts using the Sysmex XN-2000 analyzer (Sysmex, Kobe, Japan), and another without anticoagulant (BD Vacutainer PST), for a serum biochemical analysis.

### 2.8. Statistical Analysis

The data analysis was performed using a per-protocol approach, including only patients who completed the study. Descriptive statistics were used to summarize both qualitative and quantitative variables. Qualitative variables were presented as absolute frequencies and percentages, while quantitative variables were expressed as means and standard deviations for normally distributed data or medians and interquartile ranges for non-normally distributed data. The normality of quantitative variables was assessed using the Shapiro–Wilk test. For group comparisons, the chi-square test was applied to categorical variables. Quantitative variables were analyzed using the Student’s *t*-test for normally distributed data, while the Mann–Whitney U test was used for non-normally distributed data. To evaluate changes within groups between visits, paired sample *t*-tests were performed for normally distributed variables, and the Wilcoxon signed-rank test was applied for non-parametric variables.

The sample size was determined using G*Power software (version 3.1.9.7, Franz, Universität Kiel, Germany). Based on an estimated effect size (Cohen’s f) of 0.70, an alpha level of 0.05, and a statistical power of 80%, the analysis indicated that a minimum of 68 participants were required, with 34 participants per treatment arm.

Statistical analysis was performed using MedCalc 14.0 statistical software (MedCalc Software, Ostend, Belgium), all tests were two-sided, and differences associated with *p* < 0.05 were considered statistically significant.

## 3. Results

### 3.1. Participant Characteristics, Group Allocation, and Study Attrition

**Figure 1:**
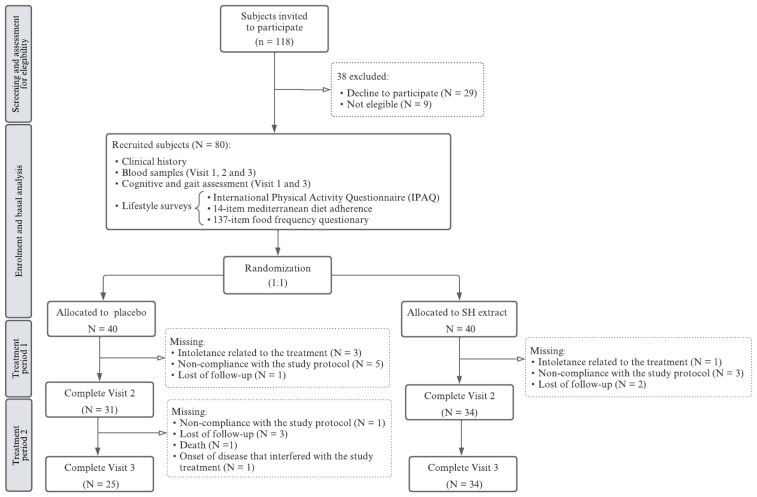
Study flow diagram.

A total of 118 individuals were initially assessed for eligibility, with 80 ultimately included and randomly assigned to either the experimental group receiving the SH extract (*n* = 40) or the control group receiving a placebo (*n* = 40). Among the 38 individuals who were excluded, 29 declined to participate and 9 did not meet the inclusion/exclusion criteria. Of the 80 participants, 65 completed the 6-month follow-up (visit 2), and 59 completed the full 11-month study period (visit 3). A total of 21 participants withdrew from the study: 15 from the placebo group and 6 from the SH extract group. Reasons for withdrawal included treatment intolerance (4 participants: 3 from the placebo group, 1 from the SH treatment group), noncompliance with the study protocol (9 participants: 6 from the placebo group, 3 from the SH treatment group), loss to follow-up (6 participants: 4 from the placebo group, 2 from the SH group), one death (placebo group), and the development of a medical condition deemed by the investigator to interfere with study participation (1 participant, placebo group) (Figure 1). 

The demographic and baseline characteristics of all participants are outlined in Table 1. The overall mean age (±SD) of participants was 68.1 ± 10.1 years, ranging from 40 to 87 years, and 31 participants (38%) were female. No statistically significant differences were observed between the treatment groups for any baseline characteristics, including age, tobacco use, and alcohol consumption, and the presence of previous medical conditions, ensuring comparability across groups. However, a significant difference was observed between groups in terms of the sex distribution (*p* = 0.012).

### 3.2. Safety Outcomes

During intervention, all AEs were recorded, but only those potentially related to the treatment were analyzed and are shown (Table 2). A total of 12 participants experienced AEs: three in the experimental group and nine in the placebo group. The most commonly reported AEs were gastrointestinal disturbances. No statistically significant differences were found in the incidence of AEs between the two groups (*p* > 0.05). Importantly, no serious AEs related to the study treatment were observed during the trial.

Regarding the recurrence of neurovascular events, two participants experienced an ischemic stroke during the intervention, with one in each experimental group. Both events were considered unrelated to the study treatment.

### 3.3. Assessment of Eating Habits and Physical Activity

Dietary habits and physical activity levels were carefully assessed using validated questionnaires. This comprehensive approach provided insight into potential lifestyle factors that could influence the efficacy of the intervention. Physical activity levels, assessed based on the IPAQ, showed that the majority of participants (61%) engaged in high levels of physical activity, with no significant differences between the placebo and SH treatment groups (Table 3). The MEDAS questionnaire, designed to assess adherence to the Mediterranean diet, revealed a mean score (±SD) of 8.7 ± 2.28. Most participants (52.54%) exhibited moderate to fair adherence to the Mediterranean diet (MEDAS score 6–9), and no significant differences were found in MEDAS score between the treatment groups. The FFQ provides detailed information on the frequency and quantity of various food items consumed, allowing for a comprehensive evaluation of participants’ dietary patterns. Results revealed no significant difference between the groups regarding total daily energy intake or macronutrient consumption (lipids, carbohydrates, and proteins). Regarding the food groups, a significant difference was found in the daily intake of pulses, with the placebo group consuming significantly more compared to the treatment group.

### 3.4. Gait Performance

To comprehensively evaluate participants’ mobility and functional performance, the gait assessment protocol incorporated multiple procedures: a measurement of spatiotemporal gait parameters, automatic gait pattern analysis, and assessment of aerobic capacity and endurance using the Six-Minute Walking Test (6MWT). At baseline, participants in the placebo and SH extract groups exhibited significant differences in several parameters (Table 4). Notably, the SH extract group had a significantly lower median height compared to the placebo group (*p* = 0.028). Similarly, at baseline (visit 1), the placebo group demonstrated significantly higher gait speed both before and after the 6MWT. Significant differences were also found in Berg’s Score and in both left- and right-hand dynamometry, with higher values in the placebo group. These results show that parameters related to the gait assessments were influenced by the unequal sex distribution between groups, with a higher percentage of men in the placebo group. Functional Ambulatory Profile (FAP) scores did not differ significantly between groups.

Gait speed before the 6MWT was significantly slower in the SH extract group compared to the placebo at baseline (*p* = 0.016), though this difference diminished after 11 months (*p* = 0.239), as occurred with Berg´s score (*p* = 0.582). The post-6MWT speed remained lower in the SH extract group (*p* = 0.011 at visit 1 and *p* = 0.065 at visit 2). In addition, the FAP score prior to the 6MWT also remained significantly higher in the placebo group after 11 months of treatment (*p* = 0.016).

Interestingly, the initial SBP and DBP, which were measured prior to the 6MWT, showed no significant differences between groups, but after 11 months (Visit 2), the SH extract group demonstrated a significantly lower SBP and DBP compared to the placebo, both before and after the 6MWT. Additionally, HR during the 6MWT revealed no significant differences between the groups at any time point.

### 3.5. Cognitive Changes

The Spanish version of the MoCA was used to determine the degree of impairment in specific cognitive domains and to detect early signs of dementia. This evaluation revealed no significant differences between the placebo and SH extract groups at either baseline or after 11 months of treatment (Table 5). Both groups showed comparable direct scores and age- and education-adjusted scaled scores with no significant improvements observed (*p* > 0.3 for all comparisons). However, when we performed intragroup analysis, the treatment group exhibited a significant improvement in both the direct and sex- and age-adjusted scalar scores of the MOCA.

### 3.6. Blood Parameters

Throughout the study, all participants maintained blood count values within the reference ranges, with no significant differences observed between treatment groups at any time point. Across the three visits, glucose levels remained relatively stable, showing no significant differences between the placebo and SH extract groups. However, a trend toward lower glucose levels was observed in the SH extract group by visit 3 (*p* = 0.084) (Table 6).

Albumin and total protein levels were generally comparable between groups, except at baseline (visit 1), where total protein levels were significantly lower in the SH extract group than in the placebo group (*p* = 0.010); this difference decreased over time. ALT and AST levels showed some variation at visit 2, with lower levels in the placebo group than in the SH extract group (ALT: 17 vs. 21.5, *p* = 0.033; AST: 21 vs. 24, *p* = 0.035). However, these differences were no longer observed after 11 months of treatment.

Significant reductions in HCys were observed in the SH extract group at both visit 2 (*p* = 0.019) and visit 3 (*p* = 0.016), indicating a potential beneficial effect on this cardiovascular risk marker. HbA1c, TSH, and other electrolytes (sodium, potassium) remained stable and comparable between groups throughout the study period.

At baseline, patients in the placebo group exhibited significantly higher total protein levels compared to the placebo group than in those assigned to treatment (7.2 vs. 7, *p* = 0.01). However, after six months of treatment (visit 2), this difference in total protein levels disappeared. At the 6-month visit, the placebo group had significantly higher homocysteine (15.4 vs. 11.5, *p* = 0.019) levels, while ALT (17 vs. 21.5, *p* = 0.033) and AST (21 vs. 24, *p* < 0.035) levels were lower compared to the treatment group. Importantly, homocysteine levels in the treatment group decreased from baseline to within the normal range (5–12 µmol/L) after six months of treatment.

When we performed an intragroup analysis, patients in the placebo group showed significant reductions in total protein, AST, ALT, and vitamin B12 after six months of treatment, alongside an increase in folic acid levels. By eleven months, total protein and vitamin B12 levels continued to decline, with additional reductions observed in potassium and homocysteine. For the treatment group, significant decreases in albumin and homocysteine were noted after six months. Further declines were observed in albumin and homocysteine at the eleven-month mark. Additionally, HDL-cholesterol and LDL-cholesterol levels decreased, while GFR, AST, and ALT levels showed a slight increase.

### 3.7. Sex-Specific Analysis of Key Variables

To better understand the treatment effects in men and women, we performed a sex-stratified analysis. Over the 11-month study period, the number of women in the placebo group decreased notably to just 4 due to the withdrawal of six participants.

When studying the sex-specific gait performance, we identified reductions in SBP and DBP both before and after the 6MWT in men and women treated with the SH extract (Table 7). These findings were consistent with the overall group analysis. Additionally, improvements in dynamometry results and Berg Balance Scale scores were noted in both sexes.

Neuropsychological assessments using the MOCA test revealed a significant improvement in direct scores after 11 months of SH extract treatment exclusively in women. In men, the observed changes did not reach statistical significance.

Biochemical analysis showed consistent trends across sexes with SH extract treatment, including a reduction in homocysteine levels and HDL-cholesterol. In men, we identified additional changes due to the treatment that aligned with findings in the whole group, such as a reduction in creatinine and an increase in GFR. Among women, a slight increase in LDL-cholesterol was noted in the stratified analysis; however, the median LDL levels remained significantly lower than those in the placebo group, suggesting limited clinical relevance due to the small sample size in the female subgroup.

## 4. Discussion

This study provides valuable insights into the effects of a polyphenol-rich *S. ramosissima* extract in patients with a history of TIA or minor stroke. To our knowledge, this is the first randomized and controlled trial to explore the safety and potential benefits of this marine plant extract in a high-neurovascular-risk population. Participants underwent an 11-month treatment period, with a comprehensive assessment of outcomes, including cognitive function, gait performance, and biochemical markers. Our findings indicate that *S. ramosissima* supplementation was associated with significant cognitive improvements, reduced homocysteine levels, and potential vascular effects, as reflected by favorable changes in LDL- and HDL-cholesterol and GFR. Notably, no serious adverse events were linked to the treatment, underscoring the supplement’s safety and tolerability over long-term use.

In our study population, dietary assessments revealed a generally moderate adherence to the Mediterranean diet among participants, with a mean MEDAS score of 8.7, consistent with studies of high-vascular-risk populations in Spain, such as the PREDIMED study [39], and others across Mediterranean countries [40].

The improvement in MOCA scores in the treated group is noteworthy. MOCA is a widely used tool for detecting mild cognitive impairment and is sensitive to changes in executive function and memory, areas commonly affected in patients with TIA or minor stroke [41,42]. Recent meta-analyses suggest potential cognitive benefits linked to polyphenol-rich foods, such as improved memory, attention, and executive functions, particularly in older adults. However, there, it emphasizes the need for further research to clarify optimal doses, specific polyphenol sources, and long-term effects on brain health [43]. Previous work on the effect of *S. europaea* L. extracts in subjects complaining of memory dysfunction did not show significant improvements in cognitive performance. However, the phenolic composition of the extract was not reported, which restricts our ability to make comparisons [33]. The improvements observed in our study could be related to the polyphenol profile of the *S. ramosissima* extract. A total of 31 polyphenolic compounds were identified in our extract, with the majority consisting of 3,5-dicaffeoylquinic acid derivatives, such as chlorogenic acid, alongside flavonoid derivatives of luteolin, quercetin, and isorhamnetin [22]. These bioactive compounds likely contributed to the observed cognitive benefits through their synergistic neuroprotective mechanisms, primarily by modulating antioxidant and anti-inflammatory pathways For instance, chlorogenic acid was shown to strengthen the gut–brain axis, reducing pro-inflammatory cytokines, and enhancing neuronal resilience via the Nrf2/PPAR pathway [44]. Luteolin derivatives regulate oxidative stress and apoptosis by modulating pathways, such as Nrf2–ARE and p53, while also crossing the blood–brain barrier to attenuate cognitive deficits [45]. Quercetin was reported to enhance cognitive function by reducing neuroinflammation, promoting neuroplasticity, and protecting neurons from apoptosis through the NF-κB, Nrf2, Akt, and AMPK pathways [46]. Similarly, isorhamnetin derivatives also mitigated neuroinflammation and enhanced synaptic plasticity by inhibiting MAPK and NF-κB signaling and activating the CREB/BDNF pathway [47]. Notably, previous research has shown cognitive benefits from polyphenol-rich extracts. In this sense, a polyphenol-rich extract from grapes and blueberries was tested in older adults with mild cognitive impairment and was associated with improvements in several cognitive tasks [48]. Additionally, previous studies have shown that chlorogenic acid was associated with significant improvements in cognitive function compared to placebo [49].

The study’s neuropsychological findings emphasize the close link between vascular health and cognitive function. The observed reduction in homocysteine levels, a known risk factor for both cardiovascular and cognitive decline [50,51], is particularly remarkable. Elevated homocysteine is linked to higher risks of ischemic events and cognitive deterioration, especially in patients with a stroke or TIA history [52,53]. The literature suggests that polyphenols may help to reduce blood homocysteine levels by promoting antioxidant activity and improving endothelial function, potentially reducing oxidative stress, which can interfere with homocysteine metabolism [54,55,56]. Certain polyphenols, such as the flavonoids present in our extract (luteolin, quercetin), are believed to support enzymes involved in homocysteine processing, which may benefit vascular health and cardiovascular risks associated with elevated homocysteine levels [57,58]. In addition, we have recently shown that Salicornia administration for 3 months can lower homocysteine levels in healthy volunteers [22]. However, more research is needed to clarify these effects in humans, and the exact mechanism through which *S. ramosissima* contributes to the homocysteine reduction remains unclear and warrants further investigation.

Interestingly, we have observed a significant reduction in SBP and DBP for both basal levels and after the 6MWT in patients receiving the SH extract for 11 months. These beneficial changes could be explained by the improvements in several factors that contribute to blood pressure regulation. In this sense, a potential link between lower homocysteine levels and better endothelial function and reduced arterial stiffness has been reported [59]. In vivo studies with extracts of *S. europaea* have demonstrated their capacity to reduce blood pressure, supporting the effects seen in our study [60]. Moreover, our results on the lipid profile suggest a potential cardioprotective role for *S. ramosissima*. The significant reductions in LDL-cholesterol and HDL-cholesterol observed in the treatment group are consistent with previous studies suggesting that marine plant extracts possess lipid-lowering properties through their rich polyphenol and antioxidant contents [61,62]. Furthermore, research indicates that quercetin and luteolin, two key components in our extracts, contribute to cholesterol reductions through multiple mechanisms. Firstly, both compounds inhibit the NPC1L1 protein, a critical mediator of cholesterol absorption, thereby decreasing intestinal cholesterol uptake. Additionally, quercetin upregulates the expression of the low-density lipoprotein receptor (LDLR), facilitating the liver’s uptake of LDL-cholesterol. Lastly, both quercetin and luteolin enhance the enzymatic activity of CYP7A1, which catalyzes the conversion of cholesterol to bile acids, promoting its excretion and further lowering cholesterol levels [63].

In addition, the observed effects on creatinine and GFR values hint at potential renal benefits, which is an area largely unexplored in prior research but could represent a new therapeutic avenue for patients at risk of chronic kidney disease after stroke [64]. These effects may be attributed to the polyphenols present in our extract, such as isorhamnetin, luteolin, and chlorogenic acid, which are known to target pathways that reduce kidney oxidative stress and inflammation [65,66,67,68,69]. Since an impaired GFR is often associated with higher blood pressure due to fluid retention and hormonal imbalances, strategies that protect kidney function indirectly help to support blood pressure control over time. Importantly, the observed increase in GFR and reduction in LDL-cholesterol are consistent with the findings from our previous study on healthy participants [22].

At baseline, the placebo group demonstrated better physical performance in gait-related parameters, likely due to an uneven gender distribution, with men typically outperforming in physical endurance tests [70]. Additionally, the placebo group’s taller average height may have contributed to their higher gait speed, observed both before and after the 6MWT [71,72]. After treatment, however, the speed disparities between groups disappeared, with the treatment group showing significant pre-6MWT speed improvements, alongside marked gains in Berg’s score and dynamometry measures on both sides.

Regarding adverse events, the recurrence of two cerebrovascular events (one in each group) is an important finding, although they were unrelated to the treatment. This is consistent with previous studies indicating that patients with a history of TIA still face a significant risk of recurrent cerebrovascular events, regardless of the specific interventions [73]. However, the absence of significant differences in adverse events between groups suggests that the *S. ramosissima* extract was well tolerated and did not cause serious side effects.

One of the key strengths of this study is the robust design—a triple-blind, placebo-controlled format, ensuring minimal bias in the reporting and interpretation of results. Additionally, the longitudinal approach, with assessments at three key points over an 11-month period, allowed for the observation of both the immediate and longer-term effects of *S. ramosissima* supplementation. Another strength is the comprehensive nature of the assessments, which included not only neuropsychological and gait performance but also a detailed biochemical analysis, offering a global view of the potential effects of the intervention. It is important to note that the population studied shares common demographic and clinical characteristics that are representative of TIA and minor stroke patients and are comparable to other large, published studies [74], which provides valuable insights into the effects of polyphenols in this particular context.

However, the study has some limitations. The relatively small sample size, exacerbated by dropout rates, may limit the generalizability of our findings. Furthermore, the imbalance in the sex distribution between the placebo and treatment groups could have influenced the physical performance outcomes, potentially confounding the interpretation of the gait results. The reduced number of women who completed the study limits our ability to draw definitive conclusions about treatment effects in this subgroup. While the observed reductions in homocysteine levels and improvements in blood pressure do not indicate sex-dependent pathways influenced by the SH extract, further investigation using larger, more balanced cohorts is warranted.

## 5. Conclusions

This study provides promising evidence that the *S. ramosissima* extract may improve cognitive function and modulate key biochemical risk factors, such as homocysteine levels, in patients with a history of TIA or minor stroke. The observed improvements in MOCA scores and blood markers related to neurovascular health suggest that this marine plant could play a role in post-TIA recovery, offering a potential natural therapeutic option for reducing the risk of recurrent vascular events and cognitive decline. While no serious safety concerns were identified and the extract was well tolerated over the 11-month period, further research is essential to clarify its underlying mechanisms. Larger, multicenter studies are necessary to confirm these findings and determine how *S. ramosissima* could be effectively integrated into clinical practice, while also exploring its specific neuroprotective and vascular effects.

## Figures and Tables

**Table 1:** Baseline characteristics of each treatment group.

	Placebo	SH Extract	*p*
	40	40	
Age (median [min; max])	70 (40–87)	69.5 (43–85)	0.242 ^1^
Male/female (*n*)	30/10	19/21	**0.012 ^2^**
Tobacco use (*n*)			0.806 ^2^
No	23	21	
Yes	4	6	
Ex	13	13	
Alcohol consumption (*n*)			0.380 ^2^
No	32	36	
Yes	6	2	
Ex	2	2	
Medical history (*n*)			
Allergies	9	13	0.317 ^2^
Intolerances	2	2	1 ^2^
Hypertension	25	27	0.639 ^2^
Diabetes	12	10	0.616 ^2^
Dyslipidemia	21	23	0.653 ^2^
Obstructive sleep apnea syndrome	3	7	0.176 ^2^
Transient ischemic attack	6	6	1 ^2^
Peripheral vascular disease	3	3	1 ^2^
Coronary disease	8	10	0.592 ^2^
Atrial fibrillation	8	8	1 ^2^
Renal insufficiency	1	1	1 ^2^

Data were analyzed using a (^1^) Student’s *t* or (^2^) Chi-square test. Bold indicates significant statistical changes.

**Table 2:** Adverse events categorized based on the MedDRA System Organ Class and Preferred Term across treatment groups.

	Placebo	SH Extract	*p*
	*N*	*N*	
Global	9	3	0.060
Gastrointestinal disorders			
Epigastralgia	1	0	0.314
Gastroesophageal reflux	1	1	1
Diarrhea	1	0	0.314
Constipation	1	0	0.314
Gas symptoms	1	1	1
Nervous system disorders			
Headache	0	1	0.314
Renal and urinary disorders			
Polyuria	3	0	0.077
Vascular disorders			
Elevated blood pressure	1	1	1
Endocrine disorders			
Blood glucose elevation	1	0	0.314
Metabolism and nutrition disorders			
Polydipsia	2	0	0.152

Data were analyzed using a Chi-square test.

**Table 3:** Physical activity level, adherence to the Mediterranean diet, and daily food group intake by participant during the 12 months prior to their inclusion in the study assessed based on the 7-item IPAQ questionnaires, 14-item Mediterranean diet adherence, and 137-item food frequency questionnaire (PREDIMED), respectively.

	Placebo 25	SH Extract 34	*p*
Physical activity level ^1^ (*n*)			0.718 ^1^
Low	5	4	
Moderate	5	5	
High	15	21	
Adherence to Mediterranean diet ^1^ (*n*)			0.064 ^1^
Low adherence (≤5)	2	3	
Medium adherence (6–9)	10	21	
High adherence (≥10)	12	6	
Daily food intake ^2^ (Median (IQR))			
Sum of vegetables (g/day)	268.2 (180–448.8)	252 (191–352.8)	0.468 ^2^
Sum of fruits (g/day)	254.8 (155.9–349.2)	274.5 (196.2–380.2)	0.651 ^3^
Sum of pulses (g/day)	24.8 (16.4–33.6)	16 (13–19.2)	**<0.001 ^3^**
Sum of cereals (g/day)	234.1 (140.8–257.5)	206.5 (132–238.8)	0.312 ^2^
Sum of whole grains (g/day)	0 (0–0)	0 (0–0)	0.975 ^3^
Sum of dairy products (g/day)	270 (215.4–447.5)	278.5 (203.5–546.2)	0.957 ^3^
Sum of meat and meat products (g/day)	114.2 (75.1–166.4)	103 (72.2–129.5)	0.412 ^3^
Total olive oil (g/day)	25 (25–50)	25 (25–33.5)	0.419 ^3^
Sum of fish (g/day)	77.4 (50.1–105.6)	64.5 (44.8–91.8)	0.247 ^3^
Sum of biscuits, cakes and sweets (g/day)	19.1 (13–29.7)	14.5 (9.2–37.2)	0.575 ^3^
Sum of industrial bakery products (g/day)	13 (3.4–21)	7 (3–30.8)	0.385 ^3^

Data were analyzed using a (^1^) Chi-square, (^2^) Student’s *t*, or (^3^) Mann–Whitney U test. Bold indicates significant statistical changes.

**Table 4:** Measurement of baseline clinical parameters before, immediately after, and at 1 and 5 min after the Six Minutes Walking Test (6MWT). Automatic measurement of gait speed and functional ambulatory profile (FAP) using the GAITRite^®^ equipment before and after the 6MWT, Berg test, and manual dynamometry. The median and interquartile range (IQR) are shown.

	Visit 1			Visit 3		
	Placebo	SH Extract	*p*	Placebo	SH Extract	*p*
	22	24		22	24	
Height (cm)	169.5 (164–173.5)	160.5 (155.5–169)	**0.028 ^2^**	–	–	-
BMI	28.3 (26.3–30.7)	29.1 (26.4–32.3)	0.375 ^2^	28.3 (26.6–29.8)	28.7 (25.7–32.7)	0.506 ^2^
SBP (mm Hg)-pre	137 (129.5–152.2)	136 (114.2–152)	0.756 ^2^	143.5 (127–150)	124 (112–136.2) *	**0.020 ^1^**
DBP (mm Hg)-pre	79 (70.2–86.5)	72 (68.5–77.5)	0.090 ^1^	77 (67.5–82.5)	70 (67.8–73.2)	**0.031 ^2^**
HR (bpm)-pre	68 (62.2–82.2)	73 (65.5–80.2)	0.538 ^1^	78 (67.5–81)	75 (65–80)	0.281 ^2^
Speed (m/s)-pre	116.8 (104.9–125.2)	91.3 (81.4–110)	**0.016 ^1^**	121.7 (106–128.9)	103.6 (83.2–124.9) **	0.2392
FAP-pre	99 (93–100)	96 (90–98.2)	0.137 ^1^	100 (96.5–100)	95 (88–98.5)	**0.016 ^1^**
6MWT (m)	481.5 (392.8–525.5)	403.5 (314.2–463)	0.066 ^1^	494 (345–524.5)	409 (359.5–489)	0.271 ^2^
Speed (m/s)-post	137.4 (118.3–146.4)	105 (84.2–123.7)	**0.011 ^2^**	128.7 (113.6–142)	107 (91.4–133.6)	0.065 ^2^
FAP-post	95.5 (87.8–98.8)	92.5 (89.8–97.5)	0.566 ^1^	96.5 (95–98.8)	95 (91.5–97.2)	0.212 ^1^
SBP (mm Hg)-post	168 (151.2–175)	148.5 (131.8–172.8)	0.098 ^2^	157.5 (139–169.8)	134.5 (126–158)	**0.029 ^1^**
DBP (mm Hg)-post	76 (73.2–79.8)	72 (66.8–78.8)	0.071 ^2^	74.5 (71.2–79.5)	73 (68–79.2)	0.260 ^2^
HR (bpm)-post	89.5 (80.5–104.2)	81.5 (77.5–103.8)	0.381 ^2^	85 (76.2–93.5)	83 (78.8–96)	0.412 ^2^
SBP (mm Hg)-post 1′	157.5 (135.2–166.8)	139.5 (122.8–163.5)	0.197 ^2^	143.5 (128.2–153.5)	125 (117.2–133.2) *	**0.010 ^1^**
DBP (mm Hg)-post 1′	76 (69.5–80.8)	71 (65–76)	**0.034 ^2^**	78.5 (72–82.8)	72.5 (68.8–77.2)	**0.022 ^2^**
HR (bpm)-post 1′	79 (72–90.2)	81.5 (71–96)	0.859 ^2^	80.5 (72.2–84.8)	78 (70–85.5) *	0.523 ^2^
SBP (mm Hg)-post 5′	135 (127.5–142.5)	132 (115–147.2)	0.695 ^2^	131.5 (120.8–146.5)	119 (108.8–124.2)	**0.009 ^1^**
DBP (mm Hg)-post 5′	78 (70.5–82.8)	71 (67.8–76)	**0.012 ^2^**	74.5 (70.2–80)	70.5 (65.2–75)	**0.026 ^1^**
HR (bpm)-post 5′	74.5 (68.5–81.5)	76 (67.5–89.2)	0.892 ^2^	74 (71.5–80.8)	75 (66.2–83.2)	0.758 ^1^
Berg’s score	56 (55–56)	54 (51–55.2)	**0.009 ^1^**	55.5 (54.2–56)	55 (53.8–56) *	0.582 ^1^
Left Dynamometry	30 (23.2–33)	16.5 (12.8–24.2)	**<0.001 ^2^**	26 (23–30)	17 (15–28.5) *	**0.019 ^1^**
Right Dynamometry	29 (21.8–31)	16.5 (13–20.5)	**<0.001 ^1^**	29 (25.5–32.8)	20 (15–30.2) **	**0.026 ^1^**

Data were analyzed using the (^1^) Mann–Whitney U or (^2^) Student’s *t* test. SBP, systolic blood pressure; DBP, diastolic blood pressure; HR, heart rate; FAP, functional ambulatory profile; 6MWT, Six minutes Walking Test. Asterisks denote significant intragroup differences compared to visit 1: * for *p* < 0.05 and ** for *p* < 0.01. Bold indicates significant statistical changes.

**Table 5:** Main results of Montreal Cognitive Assessment (MOCA) test. The median and interquartile range (IQR) are shown.

	Visit 1			Visit 3		
	Placebo	SH Extract	*p*	Placebo	SH Extract	*p*
	22	23		22	23	
MOCA						
Direct Score	22 (20.2–24.5)	20 (17.5–24)	0.355 ^2^	23.5 (21.2–26)	23 (20.5–26) **	0.793 ^1^
Adjusted SS (Age and Education)	9 (7–11)	8 (6.5–10.5)	0.486 ^1^	10 (8–12)	10 (7–11.5) *	0.577 ^2^

Data are presented as the median and interquartile range (IQR). Analyses were conducted using either (^1^) the Mann–Whitney U test or (^2^) Student’s *t*-test, and *p*-values are provided. Asterisks denote significant intragroup differences compared to visit 1: * *p* < 0.05, ** *p* < 0.01. SS: Scaled score.

**Table 6:** Biochemical profile at baseline (visit 1) and after six (visit 2) and eleven (visit 3) months of treatment by treatment group.

		Placebo	SH Extract	*p*
Glucose (mg/dL)	Visit 1	96 (86–112)	101 (92–113.8)	0.293 ^1^
	Visit 2	103 (91–112)	95 (89.2–107)	0.272 ^1^
	Visit 3	110 (93–118)	93 (86.2–108)	0.084 ^1^
Albumin (g/dL)	Visit 1	4.4 (4.3–4.6)	4.4 (4.3–4.6)	0.500 ^2^
	Visit 2	4.4 (4.3–4.6)	4.4 (4.1–4.5) **	0.238 ^2^
	Visit 3	4.3 (4.3–4.5)	4.4 (4.2–4.5) *	0.673 ^2^
Total protein (g/dL)	Visit 1	7.2 (7–7.4)	7 (6.7–7.2)	**0.010 ^2^**
	Visit 2	7 (6.8–7.2) **	6.9 (6.5–7.1)	0.113 ^1^
	Visit 3	6.9 (6.6–7.1) **	6.8 (6.6–7.1) **	0.601 ^2^
Urea (mg/dL)	Visit 1	35 (28–43)	38.5 (32.2–44.5)	0.094 ^2^
	Visit 2	36 (32.5–42)	36 (30.2–44)	0.776 ^1^
	Visit 3	37 (30–41)	36 (32–46.2)	0.630 ^2^
Creatinine (mg/dL)	Visit 1	0.9 (0.7–1.1)	0.9 (0.7–1)	0.866 ^1^
	Visit 2	0.9 (0.7–1)	0.8 (0.7–1)	0.307 ^1^
	Visit 3	0.9 (0.8–1)	0.8 (0.7–1) **	0.163 ^1^
GFR (ml/min)	Visit 1	86 (71–95)	86.5 (70.8–94.8)	0.890 ^1^
	Visit 2	87 (65–91)	85.5 (70–98.8)	0.645 ^1^
	Visit 3	82 (72–92)	86.5 (75.5–96.8) *	0.630 ^2^
Cholesterol (mg/dL)	Visit 1	149 (112–180)	133 (117.2–159.5)	0.304 ^1^
	Visit 2	151 (115–173)	137.5 (115.2–169.5)	0.804 ^2^
	Visit 3	161 (115–184)	126.5 (112.2–167.5)	0.304 ^1^
HDL (mg/dL)	Visit 1	52 (42–57)	49.5 (44.2–59.8)	0.989 ^2^
	Visit 2	52 (41–57.8)	49 (43–53.8)	0.579 ^1^
	Visit 3	46 (41–56)	43.5 (38–54) **	0.390 ^1^
LDL (mg/dL)	Visit 1	76.5 (53–94.5)	66 (54.8–74.8)	0.294 ^1^
	Visit 2	74.5 (60–88.5)	70.5 (55.8–91.2)	0.890 ^2^
	Visit 3	82.5 (55–101)	65 (54–97) *	0.477 ^1^
TGs (mg/dL)	Visit 1	98 (73–117)	84.5 (69.5–110.8)	0.421 ^1^
	Visit 2	84 (76.5–121)	85.5 (73–108.5)	0.660 ^1^
	Visit 3	83 (70–143)	85.5 (62.8–111.8)	0.452 ^1^
GGT (U/L)	Visit 1	25 (19–38)	23 (16–33.8)	0.200 ^1^
	Visit 2	23 (18.5–33)	23.5 (17.2–37.5)	0.858 ^1^
	Visit 3	27 (21–37)	21 (18–36)	0.222 ^1^
AST (U/L)	Visit 1	24.5 (19–27)	22 (18–24)	0.278 ^1^
	Visit 2	21 (17–22) *	24 (19–29.5)	**0.035 ^1^**
	Visit 3	24 (18–30)	25 (21.2–33.8) **	0.089 ^1^
ALT (U/L)	Visit 1	22 (15–26)	18 (14.2–25.8)	0.673 ^1^
	Visit 2	17 (15–22) *	21.5 (16–29.2)	**0.033 ^1^**
	Visit 3	18 (15–30)	23.5 (17.2–27.8) *	0.177 ^1^
Sodium (mEq/L)	Visit 1	142 (140–143)	141.5 (140.2–143)	0.840 ^1^
	Visit 2	141 (139–143)	141 (140–142)	0.560 ^2^
	Visit 3	142 (141–142)	140 (139.2–142)	0.103 ^2^
Potassium (mEq/L)	Visit 1	4.6 (4.5–4.8)	4.5 (4.3–4.7)	0.123 ^2^
	Visit 2	4.6 (4.3–4.7)	4.4 (4.1–4.7)	0.207 ^1^
	Visit 3	4.3 (4.2–4.7) **	4.4 (4.1–4.7)	0.831 ^2^
Vitamin B12	Visit 1	321 (262–429)	343 (247.5–413)	0.860 ^1^
	Visit 2	318 (238.2–377.8) *	346.5 (246.2–449.8)	0.279 ^1^
	Visit 3	306 (208–373) **	361 (260–478)	0.118 ^1^
Folic acid	Visit 1	5.1 (3.8–7.4)	6.4 (5.1–7.4)	0.135 ^1^
	Visit 2	6.2 (4.3–9.5) **	6.2 (4.7–8)	0.699 ^1^
	Visit 3	5.2 (3.9–7.3)	6 (4.9–9)	0.102 ^1^
HCys (µmol/L)	Visit 1	16.6 (12.8–20)	14.4 (12.5–17.1)	0.172 ^1^
	Visit 2	15.4 (12.3–18.6)	11.5 (9.6–16.8) **	**0.019 ^1^**
	Visit 3	14.6 (12.1–17.7) **	10.6 (8.7–13.7) **	**0.016 ^1^**
HbA1c (%)	Visit 1	6 (5.6–6.4)	5.8 (5.6–6.2) *	0.360 ^1^
	Visit 2	6 (5.5–6.4)	5.8 (5.5–6.4)	0.619 ^1^
	Visit 3	5.9 (5.7–6.5)	5.8 (5.6–6.3) *	0.564 ^1^
TSH (U/mL)	Visit 1	1.4 (1–2.6)	1.6 (1.2–2)	0.629 ^1^
	Visit 2	1.7 (1.1–2.6)	1.5 (1.2–2.3)	0.439 ^1^
	Visit 3	1.5 (1.2–2.5)	1.5 (1.1–1.9)	0.495 ^1^

Data are presented as the median and interquartile range (IQR). Differences between placebo and treatment groups were analyzed using either (^1^) the Mann–Whitney U test or (^2^) Student’s *t*-test, with *p*-values provided. Asterisks denote significant intragroup differences compared to visit 1: * *p* < 0.05, ** *p* < 0.01. Abbreviations: GFR, glomerular filtration rate; HDL, high-density lipoprotein; LDL, low-density lipoprotein; GGT, gamma glutamyltransferase; AST, aspartate aminotransferase; ALT, alanine aminotransferase; HbA1c, glycosylated hemoglobin; HCys, homocysteine; TGs, triglycerides; TSH, thyroid-stimulating hormone. Bold indicates significant statistical changes.

**Table 7:** Sex-specific analysis of treatment effects showing gait performance, MOCA test results, and biochemical profile at baseline (visit 1) and after eleven months of treatment (visit 3).

		Men			Women		
		Placebo	Extract	*p*	Placebo	Extract	*p*
		Median (IQR)	Median (IQR)		Median (IQR)	Median (IQR)	
Gait performance (n)		18	11		4	13	
Height (cm)		171.5 (166.2–174.8)	169 (165–174)	0.731 ^2^	156.5 (153–160.2)	158 (152–160)	0.824 ^2^
SBP (mm Hg)-pre	Visit 1	137 (128.2–152.2)	144 (127–152)	0.828 ^2^	143 (134.8–154.8)	133 (112–151)	0.545 ^2^
	Visit 3	145 (126.2–151)	117 (112–136) *	0.562 ^2^	140 (135–144)	124 (112–136)	**0.004 ^1^**
DBP (mm Hg)-pre	Visit 1	76 (70–86)	73 (71–76)	0.928 ^1^	82 (78.2–87.2)	70 (65–79)	**0.015 ^2^**
	Visit 3	73 (66.2–80.5)	72 (68–74.5)	**0.048 ^1^**	81 (78.2–83.5)	70 (67–70)	0.444 ^2^
Speed (m/s)-pre	Visit 1	116.8 (105.8–123.2)	104.3 (88.6–127.2)	0.550 ^1^	122.2 (104.6–146.9)	87.5 (76.2–98.8)	**0.013 ^2^**
	Visit 3	121.7 (106–128.2)	113.8 (92.6–151.9) *	0.751 ^2^	124.4 (108.7–136.8)	97.1 (76.3–105.2)	0.096 ^2^
FAP-pre	Visit 1	99 (93–100)	96 (94.5–98)	0.421 ^1^	97 (87.5–100)	91 (89–99)	0.608 ^1^
	Visit 3	100 (98–100)	92 (88–96.5)	**0.014 ^1^**	97.5 (95.2–99.2)	96 (89–100)*	0.646 ^1^
Speed (m/s)-post	Visit 1	137.4 (117.5–144.3)	112.8 (101.2–140.4)	0.356 ^2^	136.6 (125.6–149.9)	99.6 (81.6–115.7)	**0.021 ^2^**
	Visit 3	126.7 (108.4–142)	97.6 (48.4–143.9)	0.056 ^2^	130.7 (124.4–139.8)	103.5 (88–125.1)	0.079 ^1^
SBP (mm Hg)-post	Visit 1	168 (148.2–176.5)	156 (144.5–175.5)	0.598 ^2^	168 (161.2–172)	136 (126–170)	0.204 ^2^
	Visit 3	162 (137.8–175.5)	135 (124–167.5)	0.230 ^2^	150.5 (143–156.8)*	134 (130–145)	0.271 ^2^
SBP (mm Hg)-post	Visit 1	158.5 (132.5–166.8)	146 (133–166)	0.805 ^1^	153.5 (148.5–160)	127 (118–163)	0.157 ^1^
	Visit 3	148 (127.2–156.2)	124 (116–135.5)	0.062 ^1^	142 (138–143.2)	126 (120–133)	0.100 ^1^
DBP (mm Hg)-post 1′	Visit 1	75 (69–79.8)	76 (69.5–79.5)	0.883 ^2^	82 (79.8–85.5)	65 (64–73)	**0.010 ^1^**
	Visit 3	75.5 (71.2–80)	77 (70.5–78)	0.374 ^2^	83 (80.5–84.5)	72 (68–74)	**0.005 ^2^**
SBP (mm Hg)-post 5′	Visit 1	135 (126.2–141)	137 (119–158)	0.402 ^2^	144 (134–155)	121 (108–146)	0.229 ^2^
	Visit 3	131.5 (118.5–147)	117 (103–123) *	**0.034 ^2^**	130.5 (126.8–135)	120 (109–125)	0.089 ^1^
DBP (mm Hg)-post 5′	Visit 1	76.5 (70–81.8)	74 (70–80.5)	0.599 ^2^	81 (78.5–86.2)	70 (63–73)	**0.001 ^2^**
	Visit 3	72.5 (70–79.2)	71 (64.5–75)	0.191 ^1^	81.5 (77.8–85)	70 (68–73)	**0.013 ^2^**
Berg’s score	Visit 1	56 (54.2–56)	55 (53.5–55.5)	0.126 ^1^	56 (55.8–56)	54 (49–55)	**0.042 ^1^**
	Visit 3	55 (54–56)	55 (54.5–56)	0.962 ^1^	56 (55.8–56)	55 (53–56)*	0.219 ^1^
Left Dynamometry	Visit 1	30.5 (27–33.5)	25 (17–28)	**0.021 ^1^**	24.5 (23–26.8)	13 (11–17)	**<0.001 ^2^**
	Visit 3	26.5 (24.2–30.8)	30 (21–40) *	0.370 ^2^	21.5 (19.2–24.8) *	15 (14–16)	**0.001 ^2^**
Right Dynamometry	Visit 1	29.5 (24.2–34.5)	22 (19–30.5)	0.072 ^2^	21.5 (20.8–24)	14 (12–16)	**<0.001 ^2^**
	Visit 3	30 (27.2–34.5)	31 (29.5–40.5) **	0.443 ^1^	23.5 (21.5–25.5)	16 (15–17)*	**0.002 ^2^**
MOCA test (n)		19	12		3	11	
Direct Score	Visit 1	22 (19.5–22)	24 (19.8–27)	0.460 ^2^	25 (24–25)	20 (16–21.5)	**0.012 ^1^**
	Visit 3	23 (21–26)	24.5 (22.5–27)	0.882 ^2^	26 (25–26)	22 (19.5–24) *	0.481 ^1^
Adjusted SS (Age and Education)	Visit 1	9 (7–11)	9.5 (5.8–11)	0.500 ^2^	11 (8–12)	8 (7–9.5)	0.143 ^2^
	Visit 3	10 (8–13)	9.5 (7–12.2)	0.853 ^2^	10 (9–10.5)	10 (7–11)	0.902 ^2^
Biochemical analysis (n)		21	18		4	16	
Creatinine (mg/dL)	Visit 1	0.9 (0.9–1.1)	0.9 (0.8–1)	0.693 ^1^	0.7 (0.6–0.7)	0.7 (0.7–0.9)	0.320 ^1^
	Visit 2	0.9 (0.8–1.1)	0.9 (0.8–1)	0.455 ^1^	0.7 (0.6–0.7)	0.8 (0.6–0.8)	0.237 ^1^
	Visit 3	0.9 (0.8–1)	0.9 (0.7–1) **	0.375 ^1^	0.8 (0.7–0.8)	0.7 (0.6–0.9)	0.705 ^1^
GFR (ml/min)	Visit 1	86 (67–95)	89.5 (74–100)	0.481 ^1^	86.5 (84–97.2)	84 (67.2–91.2)	0.368 ^1^
	Visit 2	85 (65–91)	92.5 (76.8–100.8)	0.163 ^1^	88 (87–97)	79.5 (70–92)	0.201 ^1^
	Visit 3	83 (71–92)	91 (77.8–103.8) *	0.162 ^2^	77.5 (74.2–89)	83 (69.8–94.2)	0.524 ^2^
HDL (mg/dL)	Visit 1	48 (40–54)	47 (44–50.8)	0.761 ^2^	55.5 (53–67)	56.5 (48.5–62)	0.670 ^1^
	Visit 2	47.5 (39.5–52.8)	48 (43–50)	0.751 ^1^	60 (57.8–69.5)	52 (42.5–60.8)	0.122 ^1^
	Visit 3	43 (41–50)	41 (38–45.5) *	0.162 ^1^	63 (58–68.5)	54 (42.2–59.8) *	0.127 ^2^
LDL (mg/dL)	Visit 1	71 (53–90)	64.5 (53–72.8)	0.422 ^1^	96 (85–105)	67 (56.2–93)	0.121 ^2^
	Visit 2	71 (57.5–86.8)	72 (58.8–89.8)	0.861 ^2^	88 (76–103)	69.5 (55.8–91.5)	0.309 ^2^
	Visit 3	68 (54.2–99.2)	63 (53.2–75.5)	0.539 ^1^	106.5 (99.8–109.2)	76 (58.5–105.5) *	0.245 ^2^
Homocysteine (µmol/L)	Visit 1	17 (15.6–20.1)	16.1 (13.8–19.2)	0.390 ^1^	12.6 (10.8–14.8)	13.4 (11.2–15)	0.813 ^1^
	Visit 2	15.6 (13.7–20.1)	11.4 (9.2–15.2) **	**0.010 ^1^**	10.8 (9–13.3) *	11.8 (9.6–17.6)	0.537 ^2^
	Visit 3	14.7 (12.2–18) **	11.5 (10.3–14.6) **	0.111 ^1^	11.6 (10.6–13.5)	9.6 (8.5–12.9) **	0.298 ^1^
HbA1c (%)	Visit 1	6.1 (5.7–6.4)	5.7 (5.4–6)	0.101 ^1^	5.5 (5.4–5.9)	5.8 (5.6–6.2)	0.235 ^1^
	Visit 2	6 (5.6–6.4)	5.8 (5.4–5.9)	0.085 ^1^	5.4 (5.4–5.8)	5.8 (5.7–6.5) *	0.218 ^1^
	Visit 3	5.9 (5.7–6.5)	5.8 (5.6–6)	0.153 ^1^	5.6 (5.5–5.9)	5.8 (5.7–6.6) *	0.256 ^1^

Data are presented as median and interquartile range (IQR). Differences between placebo and treatment groups were analyzed using either the (^1^) the Mann–Whitney U test or (^2^) Student’s *t*-test, with *p*-values provided. Asterisks denote significant intragroup differences compared to visit 1: * *p* < 0.05, ** *p* < 0.01. Abbreviations: SBP, systolic blood pressure; DBP, diastolic blood pressure; FAP, functional ambulatory profile; SS, scaled score; GFR, glomerular filtration rate; HbA1c, glycosylated hemoglobin. In the variables of gait performance, those ending with “-pre” and “-post” refer to measurements taken before and after the Six-Minute Walking Test (6MWT). Bold indicates significant statistical changes.

## Data Availability

Data will be made available on request. The data are not publicly available due to being a part of an ongoing study.
